# Bilateral Medial Medullary Stroke: A Challenge in Early Diagnosis

**DOI:** 10.1155/2013/274373

**Published:** 2013-10-02

**Authors:** Amir M. Torabi

**Affiliations:** East Dallas Neurological Services Baylor Medical Center, Garland, TX, USA

## Abstract

Bilateral medial medullary stroke is a very rare type of stroke, with catastrophic consequences. Early diagnosis is crucial. Here, I present a young patient with acute vertigo, progressive generalized weakness, dysarthria, and respiratory failure, who initially was misdiagnosed with acute vestibular syndrome. Initial brain magnetic resonance imaging (MRI) that was done in the acute phase was read as normal. Other possibilities were excluded by lumbar puncture and MRI of cervical spine. MR of C-spine showed lesion at medial medulla; therefore a second MRI of brain was requested, showed characteristic “heart appearance” shape at diffusion weighted (DWI), and confirmed bilateral medial medullary stroke. Retrospectively, a vague-defined hyperintense linear DWI signal at midline was noted in the first brain MRI. Because of the symmetric and midline pattern of this abnormal signal and similarity to an artifact, some radiologists or neurologists may miss this type of stroke. Radiologists and neurologists must recognize clinical and MRI findings of this rare type of stroke, which early treatment could make a difference in patient outcome. The abnormal DWI signal in early stages of this type of stroke may not be a typical “heart appearance” shape, and other variants such as small dot or linear DWI signal at midline must be recognized as early signs of stroke. Also, MRI of cervical spine may be helpful if there is attention to brainstem as well.

## 1. Introduction

Bilateral medial medullary stroke is very rare, and clinical diagnosis without neuroimaging is very difficult [1]. Brainstem encephalitis and Guillain-Barre's syndrome (GBS) can present similarly [2]. Despite a huge progress in MRI technology, still human factor and experience can determine correct interpretation. Here, I discuss a clinical case and MRI findings of a patient with this diagnosis.

## 2. Case Presentation 

A 59-year-old white male patient, right-handed, presented with acute vertigo, nausea, and vomiting to a University Hospital in Dallas, TX, USA, on April 17, 2013. Past medical history was remarkable for untreated hypertension and moderate alcohol consumption. He was not taking any medication at home. Initial examiner (an internist) in that hospital noted bilateral nystagmus, although he did not specify direction or other characteristics of the nystagmus. 

**Figure 1:**
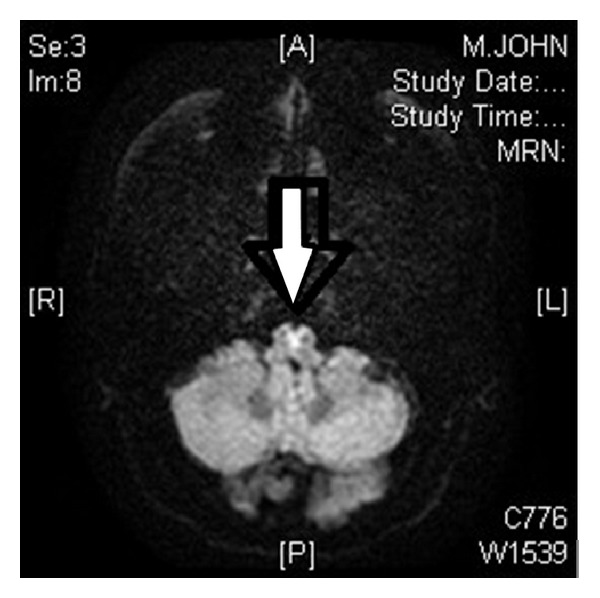
MRI of brain and DWI at presentation. Abnormal signal at DWI, a midline dot-linear signal, was similar to an artifact.

**Figure 2:**
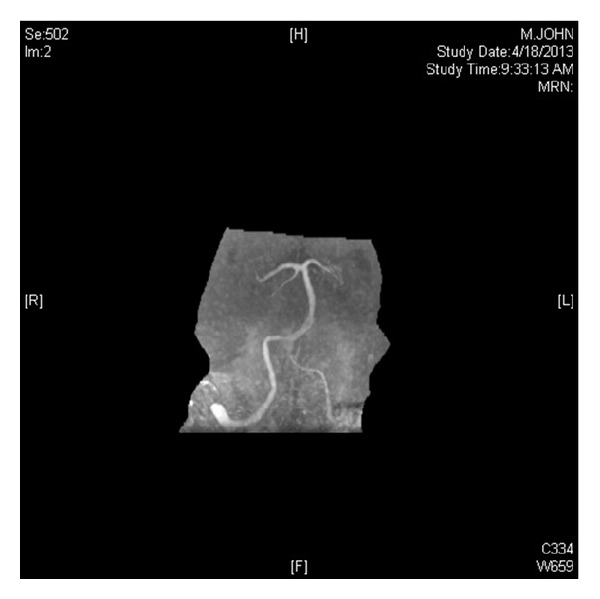
MRA of brain does not show any abnormality of vertebral arteries.

MRI of brain was done and read as normal (Figure 1), although a faint linear signal at DWI at midline medulla could be seen retrospectively. MR angiography of head (Figure 2) and neck was normal. Patient was told may have acute labyrinthitis and was discharged home two days later. After discharge his condition slowly deteriorated, and he presented to our hospital, Baylor Medical Center at Garland, TX, USA, three days later with generalized weakness, falls, slurred speech, respiratory failure, and dysphagia. Initially he was intubated due to risk of aspiration. His evaluation after extubation showed severe dysarthria with normal comprehension. His cranial nerves revealed direction changing gaze evoked horizontal nystagmus. No facial palsy noted, and extraocular movement still was full. Hypoglossal diplegia as well as decreased gag reflex was noted. Motor exam showed diffuse bilateral weakness of both upper and lower extremities at the range of 2-3/5, along with decreased tone and absent deep tendon reflexes everywhere, similar to an acute spinal cord shock state. He had mute Babinski response bilaterally. Shortly after examination, he developed respiratory failure and was reintubated.

**Figure 3:**
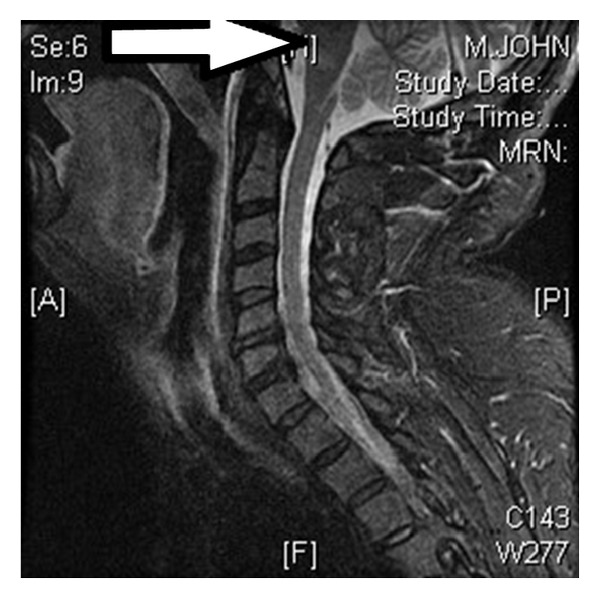
MRI of cervical spine, sagittal, shows abnormal T2 hyperintense lesion at mid-upper medulla.

**Figure 4:**
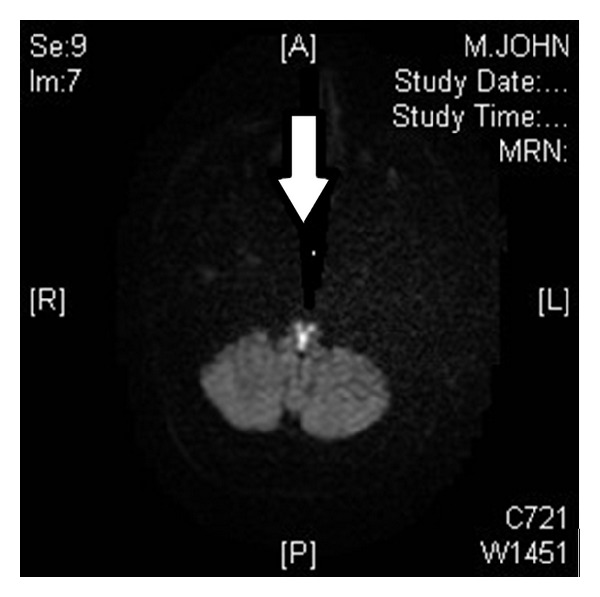
Second brain MRI, DWI shows characteristic “heart appearance” sign suggestive of bilateral medial medullary infarct.

 For the possibility of GBS and brainstem encephalitis, lumbar puncture was performed which showed normal cell, protein, and glucose. Blood and CSF cultures were negative. Since GBS still could not be excluded, he was started on empiric intravenous immunoglobulin (IVIG). Thiamine 100 mg intravenous was given for the possibility of Wernicke's encephalopathy, and Aspirin 325 mg per day was added for the possibility of stroke. MRI of cervical spine (Figure 3) was ordered to make sure that there was no high cervical cord lesion. Cervical cord was unremarkable, but interestingly, a T2-hyperintense lesion was noted at midanterior medulla (Figure 3). Because of this finding, a repeat MRI of brain was requested (Figure 4), which confirmed bilateral anterior medial medullary stroke, typical “heart appearance sign.” IVIG was stopped, and he was put on full dose enoxaparin 1 mg/kg twice a day, but there was progressive weakness to the point of quadriplegic state in less than two days. Still he was able to communicate with eye movements and blinking. He had tracheostomy and remained ventilator dependent for another five days, then he decided to stop his ventilator, and shortly after he died of respiratory failure. 

## 3. Discussion

Bilateral medial medullary stroke is a very rare type of stroke. The most common symptoms are weakness, dysarthria, hypoglossal palsy, flaccid, or spastic quadriplegia. It has generally a poor prognosis [1, 3, 4]. The characteristic brain MRI finding of “heart appearance” at DWI has been described in multiple case reports [1, 3, 4]. In this case, the first MRI (Figure 1) done in acute phase did show a small linear abnormal DWI signal at midline but was missed by radiologist and clinician. 

There have been other case reports of this type of stroke, missed by radiologists. Considering interventions such as thrombolytic in the acute phase, it is important to diagnose any stroke as early as possible, and such subtle finding needs to be picked up by radiologist or neurologist for an effective treatment. Normal MRA of brain may suggest small vessel disease of one of the paramedian perforatings (from vertebral or anterior spinal arteries). It is possible if one paramedian artery is supplying both pyramids in rare cases.

Brainstem encephalitis is also very rare and has a broad range of differential diagnosis including infectious and autoimmune causes. It has features such as ophthalmoparesis, ataxia, weakness, and upper motor neuron signs. In this case, negative CSF, absence of ophthalmoplegia, and MRI findings are all against brainstem encephalitis. GBS similarly can mimic the presentation, particularly with diffuse areflexia and progressive weakness, and was excluded by MRI findings [2]. MRI of cervical spine was helpful to view abnormal signal at upper medulla. Sagittal FLAIR brain MRI is equally helpful but may not be done routinely. 

 Overall outcome of this type of stroke is not good with severe morbidity and mortality, and early diagnosis based on combination of clinical and MRI findings by both neurologist and radiologist is critical. MRI findings particularly in the first few hours may not be typical, and along with clinical presentation neurologists and radiologists should have a high index of suspicion.
